# Knockdown of SPON2 inhibits the growth of triple-negative breast cancer

**DOI:** 10.3389/fonc.2023.1141417

**Published:** 2023-03-06

**Authors:** Xueyi Hu, Caiwu Su, Jian Wei

**Affiliations:** Sinopharm Dongfeng General Hospital, Hubei University of Medicine, Shiyan, Hubei, China

**Keywords:** SPON2, TNBC, ShRNA, transcriptome sequencing, cancer

## Abstract

**Objective:**

Spondin-2 (SPON2) is highly expressed in a variety of tumors and has been associated with poor prognosis, but the relationship to triple-negative breast cancer (TNBC) is unclear. The aim of this study is to investigate the expression of SPON2 in TNBC and its function.

**Methods:**

Immunohistochemistry was used to detect the expression of the SPON2 protein in TNBC and in normal tissue adjacent to cancer and breast fibroadenoma. The GEO database GSE76275 dataset was used to study the expression of SPON2 mRNA in TNBC and non-TNBC. ​The expression of SPON2 mRNA was detected by qPCR in TNBC cells MDA-MB-231, non-TNBC breast cancer cells MCF-7, and normal breast cells MCF-10A. ​Kaplan Meier-Plotter database was used to analyze the relationship between SPON2 expression and TNBC prognosis. ​ShRNA lentivirus was used to knock down high expression of SPON2 in TNBC cells. The effects of knockdown of SPON2 expression on the proliferation, migration, invasion, apoptosis, and subcutaneous tumorigenic ability of TNBC cells in nude mice were analyzed using CCK8, clone formation assay, scratch assay, transwell migration assay, transwell invasion assay, Hoechst apoptosis assay, and tumorigenic ability in nude mice. Transcriptome sequencing of TNBC cells with knockdown SPON2 expression. ​In combination with the GEO database, GO and KEGG analyses were performed, and psychophysiological interaction Protein-Protein Interaction Networks (PPI) analysis was performed for transcriptome sequencing of the differentially expressed genes. ​The changes in the expression of PI3K-ATK pathway proteins after SPON2 knockdown were detected by Western blot.

**Results:**

Our study shows that upregulation of SPON2 in TNBC is associated with poorer patient outcomes. Knockdown of SPON2 inhibited TNBC cell proliferation, clone formation, migration, invasion, and tumorigenic ability and promoted apoptosis. Knockdown of SPON2 up-regulated TNBC cell adhesion and down-regulated PI3K-ATK pathway, and PPI results showed that CCL2 was the key protein.

**Conclusions:**

SPON2 may be a valuable biomarker for the diagnosis and prognosis of TNBC and is a potential therapeutic target for TNBC.

## 1 Introduction

Recently published data show that breast cancer is now the most prevalent malignancy worldwide and, globally, it is the most common type of malignancy and the leading cause of cancer death in women (1). Triple-negative breast cancer (TNBC) refers to breast cancer with negative ER (estrogen receptor), PR (progesterone receptor), and HER2 (human epidermal growth factor receptor 2) expression; TNBC has a high incidence, accounting for 10–20% of breast cancers (2). TNBC is a heterogeneous and aggressive group of diseases with a higher risk of recurrence, metastasis and death compared with other breast cancer subtypes (3). Due to the unique biological behavior of TNBC and its insensitivity to endocrine therapy and conventional anti-HER2-targeted therapy, chemotherapy is still considered the main treatment for TNBC, but a significant proportion of patients are clinically resistant to chemotherapy (4). Therefore, it is important to find targets for precise treatment of TNBC and more effective treatment modalities.

Many factors are involved in tumor progression and metastasis, among which the tumor microenvironment plays an important role. The extracellular matrix is an important component of the tumor microenvironment, through which tumor cells can enhance their motility and invasive ability and can undergo distant metastasis through the remodeled extracellular matrix (5, 6).

SPON2 (Spondin2), alias M-spondin, or DIL-1 is a member of the secreted extracellular matrix protein Mindin-F-spondin family; the human-derived SPON2 protein consists of 331 amino acid residues and has a molecular weight of approximately 36 KD (7, 8). SPON2, as an extracellular matrix, is involved in the innate immune response (9). SPON2 has multiple functions such as recruitment of inflammatory cells and activation of the intrinsic immune response (10, 11).

In recent years, there has been a gradual increase in the number of studies on the relevance of SPON2 to tumors. Recent studies have shown that SPON2 protein is highly expressed in lung cancer, colorectal cancer, liver cancer, and other tumors and is expected to be a new therapeutic target for cancer (12–14). SPON2 promotes the proliferation, migration, and invasion of gastric cancer cells (10). Silencing SPON2 reduces the growth and proliferation capacity of mouse gastric cancer cells, whereas overexpression of SPON2 enhances the growth and proliferation capacity of tumor cells with increased activity (15). Knockdown of SPON2 gene was able to inhibit the invasive and migratory ability of renal clear cell carcinoma cells (16). There are no reports on the expression of SPON2 in TNBC and the role it plays.

This study confirms that SPON2 is up-regulated in TNBC and is associated with poor patient prognosis. Knockdown of SPON2 inhibited TNBC cell proliferation, clone formation, migration, invasion, and tumorigenic ability in nude mice and promoted apoptosis. SPON2 may play a role in TNBC through cell adhesion, PI3K-AKT pathway.

## 2 Materials and methods

### 2.1 Sample source

Twelve cases of surgically resected paraffin specimens of TNBC archived at the Department of Pathology of the State Pharmaceutical Dongfeng General Hospital affiliated to the Hubei Medical College from 2018-01-01 to 2022-05-01 were randomly selected for the study, as well as the corresponding normal specimens of TNBC adjacent to cancer (at least 5 cm from the tumor margin) and 20 cases of breast fibroadenoma, which were pathologically confirmed, and all patients were not combined with other malignant tumors and were not treated with preoperative radiotherapy.

### 2.2 Data source and description

The GSE76275 (17) dataset was downloaded from the GEO (https://www.ncbi.nlm.nih.gov/geo/query/acc.cgi) database, which contains 198 TNBC cases and 67 non-TNBC samples. Download the dataset of GSE103091 (18), which contains 238 TNBC samples.

SPON2 mRNA expression data were obtained by processing GSE76275 and GSE103091 using the tidyverse, limma, and hgu133plus2.db packages in the R language. Differences in SPON2 mRNA expression in TNBC and non-TNBC in GSE76275 were analyzed and plotted using the software GraphPad Prism.

### 2.3 Immunohistochemistry

Paraffin specimens of TNBC cancer and adjacent normal paraffin specimens of cancer and breast fibroadenoma were taken out and preserved in the pathology department. The slices were cut and placed in an oven at 60°C for 3h, dewaxed in xylene and placed in citric acid antigen repair solution for thermal repair, incubated with H2O2 for 10 min at room temperature, incubated dropwise with SPON2 rabbit polyclonal antibody (Proteintech, 20513-1-AP, dilution 1:500) overnight at 4°C in the refrigerator; drives with horseradish peroxidase-labeled Goat anti-rabbit secondary antibody (GeneTex, No. GTX213110-01, dilution 1:500) was added dropwise and incubated for 30 min at room temperature; the smear was developed with chromogenic agent (DAB) for 8 min at room temperature and then rinsed with tap water for 1 min; hematoxylin was used for re-staining, gradient ethanol was used for dehydration, and the smear was sealed. PBS was used instead of primary antibody as negative control.

Five fields of view were randomly selected for scoring using a microscope (×400). The percentage of positive cells ≤ 5% = 0 points, 5–25% = 1 point, 25–50% = 2 points, 50–75% = 3 points, 75% or more is 4 points; observe the intensity of the stain, no stain = 0 points, weak stain = 1 points, moderate intensity stain annotation = 2 points, strong stain = 3 points. Combining the two results, the product of the percentage of positive cells and staining intensity was used as the final score.

### 2.4 Kaplan-Meier Plotter analysis of the relationship between SPON2 expression and clinical prognosis of TNBC

The relationship between SPON2 expression levels and the prognosis of TNBC patients was analyzed using the Kaplan-Meier Plotter (http://kmplot.com/analysis/) database. In the “gene chip,” select “start KM Plotter for breast cancer,” retrieve the “SPON2” gene, set ER, PR, and HER2 as negative in the restriction conditions, and plot the RFS curve related to the prognosis of TNBC and “SPON2” expression. Finally, GO analysis and KEGG analysis were performed, and the top 6 results in this study were selected in ascending order of *P*-value (*P* < 0.05).

### 2.5 Functional enrichment analysis

Pearson correlation analysis was performed on the datasets GSE76275 and GSE103091 downloaded from GEO, and the 500 genes with *R* > 0 and most correlated with SPON2, respectively, were selected and uploaded to DAVID (https://david.ncifcrf.gov/), where the official gene names were selected as identifiers and species selected for Homo sapiens.

### 2.6 Cell culture

Human breast cancer cell line MDA-MB-231 cells and MCF-7 cells were cultured in Dulbecco's Modified Eagle Medium (DMEM) medium containing 10% fetal bovine serum, and human breast normal cells MCF-10A were cultured in saturated humidity at 37°C and 5% incubator using MCF-10A special medium.

### 2.7 Lentiviral transfection

​The lentiviral vector carries features such as a green fluorescence protein gene and puromycin resistance, and successfully transfected cells are able to express green fluorescence normally and are resistant to puromycin. Using the 1/2 small volume infection method, 1/2 volume of fresh culture fluid was added at the time of virus infection and replenished to the culture volume after 4h of lentivirus infection. MDA-MB-231 cells at logarithmic growth stage were taken, digested and counted, then diluted to 3*105/ml and inoculated with 12-well plates, with the number of cells per well-being approximately 3*105. Place in 37°C, 5% incubator and incubate overnight. The next day, cell confluence ranged between 30 and 50%. The original medium was discarded, 500 µl of fresh medium was added, and ShRNA-SPON2 lentivirus and ShRNA-Ctrl control virus were added for infection according to the pre-experimental mapping multiplicity of infection (MOI) of 20. ShRNA-SPON2 sequence is CAGGGACAATGAGATTGTAGA (10) and ShRNA-Ctrl sequence is TTCTCCGAACGTGTCACGTAA. The interfering vector selected was HBLV-U6-MCS-CMV-ZsGreen-PGK-PURO. A working concentration of 4 µg/ml of polythene was added to increase the efficiency of the infection, and 500 µl of the medium was replenished after 4h. After 24h of virus addition, the culture medium containing the virus was discarded and replaced with fresh complete medium to continue the culture. After 72h of infection, the infection efficiency was observed by fluorescence microscopy. After the cell state was stabilized, the cell line was switched to a working concentration of 0.5 µg/ml of Puromycin to screen for stable transfection.

### 2.8 Quantitative polymerase chain reaction

Logarithmic growth phase cells were taken, total RNA was obtained by total RNA extraction kit, and the concentration and purity of total RNA were measured by spectrophotometer. Samples with OD260/OD280 between 1.8 and 2.0 were assayed with a one-step reverse transcription-fluorescence quantification kit. The primers were synthesized by Shanghai sangon biotech, and the upstream and downstream primer sequences are shown in the **Table 1**. The quantitative polymerase chain reaction (qPCR) reaction conditions were 50°C for 30 min (reverse transcription), 95°C for 3 min (pre-denaturation), 95°C, 15 s (40 cycles), 60°C, and 30 s (40 cycles). Three replicate wells were set for each sample, and the average CT value was taken as the final CT value of that sample. Relative gene mRNA expression expressed as 2^-ΔΔCT^

**Table 1:** qPCR primer sequence.

Gene	Primer sequences	
SPON2	forward	5´-GATTGTAGACAGCGCCTCAGTTCC-3´
	reverse	5´-GACGCACTCAGCCTCTTCTTCG-3´
ALPK2	forward	5´-TCCGAAGGACCAGGGACTCTAT-3´
	reverse	5´-CGGTGAACCCCTTCTCCAAA-3´
LGALS12	forward	5´-GCCTGGGCAGGTCATCATAG-3´
	reverse	5´-GAGTTCTGTCTGCGAAGGAGG-3´
OLR1	forward	5´-CTTTGGATGCCAAGTTGCTGAA-3´
	reverse	5´-GCATCAAAGGAGAACCGTCC-3´
TRPV4	forward	5´-TGGCTTCTCGCATACCGT-3´
	reverse	5´-GGCTCTGGCGTTGGCTTA-3´
β-actin	forward	5´-ACTATCGGCAATGAGCGGTTCC-3´
	reverse	5´-CTGTGTTGGCATAGAGGTCTTTACG-3´

### 2.9 Western blot assay

Protein was collected from each group of logarithmic growth phase cells, protease inhibitor and phosphatase inhibitor were added, protein concentration was measured by BCA method, and boiled at 100°C for 5 min, 30 µg of equal amount of protein was firstly separated by SDS-PAGE electrophoresis and then transferred to PVDF membrane. BSA (5%) was closed at room temperature for 1h. Rabbit anti-human polyclonal antibodies SPON2 (1:500), AKT (1:1000), P-AKT (1:1000), PI3K (1:1000), P-PI3K (1:1000), and β-actin (1:1000) were added and incubated overnight at 4°C, respectively. After TBST washing, HRP-labeled secondary antibody (1:5000) was added, incubated at room temperature for 1h. After TBST washing, ECL luminescent solution was developed and exposed, the film was scanned, and the grayscale values of the bands were measured on Image J analysis software to calculate the relative protein content.

### 2.10 CCK8

Cells in logarithmic phase were taken from each group, digested and counted, 2 × 10three cells (100 µl) were added to each well in a 96-well plate, and sterile PBS solution was added to the edge wells. Five parallel control wells were set up for each group of cells, and the cell-free medium wells were used as zeroing wells, and a total of five plates were planted. The culture was incubated at 5% CO2 and 37°C. One plate was removed every 24h for measurement, and 10 µl of CCK8 was added to each well and incubated at 37 °C for 2h. The OD value of 450 nm was measured by enzyme marker.

### 2.11 Colony formation assay

Cells of each group in logarithmic growth phase were taken, digested, counted, inoculated in six-well plates 200 per well, and three wells of each group were repeatedly inoculated and cultured at 5% CO2 and 37°C, and the solution was changed every 3 days. After 20 days, the culture was terminated, the medium was discarded, washed twice with PBS, fixed in 95% alcohol for 10 min, then stained with 0.5% crystal violet for 20 min, washed with PBS, and photographed.

### 2.12 Wound-healing assay

Cells of each group in logarithmic phase were inoculated in six-well plates and cultured at 5% CO2 and 37°C. When cell fusion grows all over the well plate, use a 200 µl gun to form scratches in the cell layer. Rinse three times with PBS to wash off the scratched cells. Serum-free DMEM medium was added for incubation and photographed at 0h and 24h after scratching.

### 2.13 Transwell migration and invasion

Cells in logarithmic phase were taken from each group, resuspended in serum-free DMEM medium and adjusted to a cell density of 5 × 105/ml, then inoculated with 100 µl per well in the upper chamber of Transwell, and 700 µl of DMEM medium containing 15% FBS was added to each well of the 24-well plate. After incubation in the incubator for 24h, the upper chamber medium was discarded, washed twice with PBS, and fixed in 95% alcohol, stained with 0.5% crystal violet, and the residual cells on the upper chamber surface were wiped off with cotton swabs, and the cells on the lower chamber surface were observed under a ×200 magnification microscope and the number of migrating cells was counted. The Matrigel was diluted 6:1 with serum-free DMEM, wrapped with 100 µl per well on the upper chamber surface of transwell bottom membrane, air-dried at 4°C, and placed in a 24-well plate, and the above steps were repeated to count the number of invading cells.

### 2.14 Transcriptome sequencing

Sh-SPON2 and Sh-Ctrl cells in log phase growth were taken, digested, centrifuged, and placed in 2-ml EP tubes, resuspended with 1 ml of TRIzol, and snap-frozen in liquid nitrogen immediately after sampling. The transcriptome was sent in sufficient dry ice in an insulated box, and transcriptome sequencing was performed by Sangon Biotech, with three biological replicates for each group. The vegan package for R is used for Principal Component Analysis. DESeq2 was used to perform differential gene expression analysis. In order to obtain genes with significant differences, we set the screening condition to *q* < 0.05 and the multiple of difference |FoldChange|>2. GO analysis and KEGG analysis were performed for differential genes using the R language clusterProfiler package, and the top 6 results were selected in ascending order of *P*-value in this study (*P* < 0.05). Protein interaction network analysis of differential genes was performed using the igraph package of R and visualized with Cytoscape software.

### 2.15 Tumor formation in nude mice

We purchased 4- to 6-week-old female nude mice (BALB/c), three in each group, and injected 3*106/each Sh-SPON2 and Sh-Ctrl cells in logarithmic growth subcutaneously in the right axilla, and after tumor formation, the length (L) and width (W) were measured by vernier calipers every 5 days, and the volume *V* = 0.52*L*W^2^. After 45 days, the nude rats were executed, and the tumors were weighed and photographed.

### 2.16 Hoechst apoptosis

When apoptosis occurs in cells, the chromatin is solidified. Therefore, after Hoechst 33258 staining, the nuclei of normal cells appear normal blue color when observed under fluorescence microscope, while the nuclei of apoptotic cells will be dense and dense stained, or fragmented and dense stained with some whitish color. We transfected the logarithmic growth phase cells with lentivirus for 72h, aspirated the culture fluid, and added 0.5 ml of 95% alcohol to fix for 10 min. We removed the fixative, washed twice with PBS or 0.9% NaCl for 3 min each time, and aspirated the liquid. We added 0.5 ml of Hoechst 33258 staining solution and stain for 5 min. We removed the staining solution and washed twice with PBS or 0.9% NaCl for 3 min each time and aspirate the liquid. Fluorescent microscopes are used to detect.

### 2.17 Statistical methods

Statistical analyses and visualization were performed in R (version 4.1.3) and IBM SPSS Statistics (version 23.0). An unpaired *t*-test tests the significance of the difference between the two groups. One-way ANOVA was performed to test the significance of differences between more than two groups. The significance of the correlation between the two groups was tested by Pearson correlation analysis. The difference was considered statistically significant at *P* < 0.05.

## 3 Results

### 3.1 SPON2 expression is up-regulated in TNBC and correlates with poor prognosis

**Figure 1:**
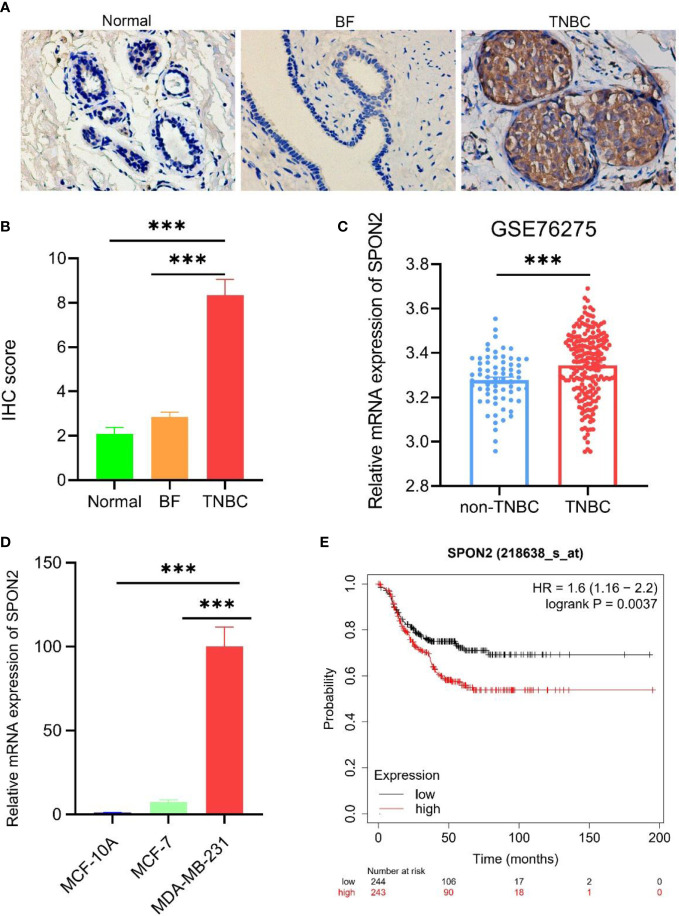
SPON2 is highly expressed in TNBC and leads to poor prognosis. **(A, B)** Immunohistochemical detection of SPON2 protein expression in TNBC, breast fibroadenoma (BF), and normal tissue adjacent to TNBC cancer showed that SPON2 protein was highly expressed in TNBC. Magnification times: ×200. **(C)** SPON2 mRNA expression levels were higher in TNBC than in non-TNBC breast cancer (non-TNBC) in the GSE76275 dataset. **(D)** The qPCR assay showed that SPON2 mRNA was highly expressed in MDA-MB-231. **(E)** High SPON2 expression in TNBC had worse recurrence-free survival. ***P < 0.001.

To investigate the expression level of SPON2 in TNBC, we performed immunohistochemical experiments on 12 cases of TNBC and its paraneoplastic normal samples and 20 cases of breast fibroadenoma, and the results showed that the expression of SPON2 was higher in TNBC than in the paraneoplastic normal tissue and breast fibroadenoma, **Figures 1A, B**. Furthermore, we analyzed the difference in SPON2 mRNA expression between 198 TNBC and 67 non-TNBC breast cancers in the GEO database GSE76275 dataset, and the results showed that SPON2 mRNA expression was higher in TNBC than in non-TNBC breast cancers, **Figure 1C**. Then, we detected the SPON2 mRNA expression levels in TNBC cells MDA-MB-231, non-TNBC breast cancer cells MCF-7, and normal breast cells MCF-10A by qPCR, which showed that SPON2 expression was higher in MDA-MB-231 than MCF-7 and higher in MDA-MB-231 than MCF-10A, **Figure 1D**.

To investigate the effect of high SPON2 expression in TNBC on its prognostic RFS, we performed an analysis using the Kaplan-Meier Plotter database, which showed that high SPON2 expression in TNBC had worse RFS, **Figure 1E**.

### 3.2 Functional enrichment analysis

**Figure 2:**
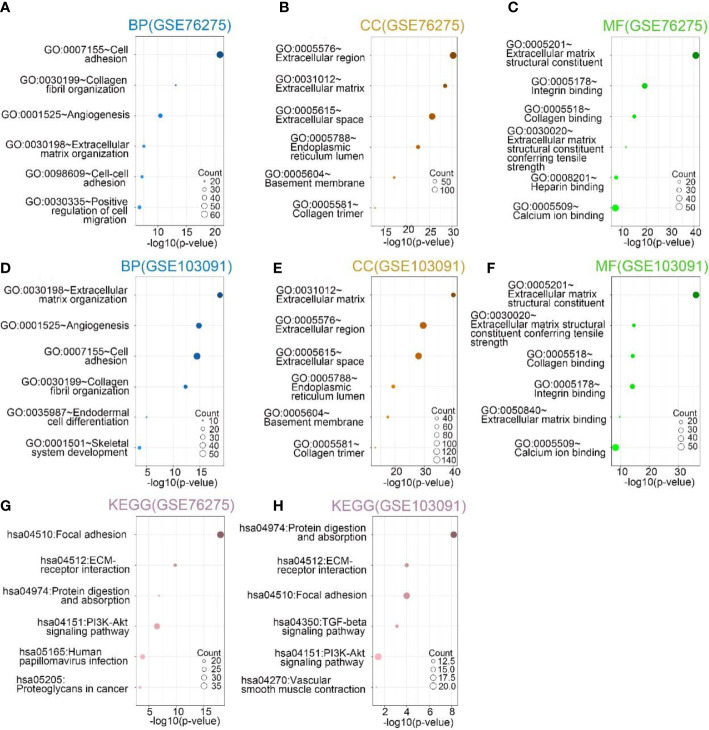
**(A–C, G)** In GSE76275, the top 6 biological processes (BPs), cellular components (CCs), and molecular functions (MFs), Kyoto Encyclopedia of Genes and Genomes (KEGG) pathway analysis most related to SPON2. **(D–F, H)** In GSE103091, the top 6 biological processes (BPs), cellular components (CCs), and molecular functions (MFs), Kyoto Encyclopedia of Genes and Genomes (KEGG) pathway analysis most related to SPON2.

To further investigate the biological functions played by SPON2 in TNBC. The genes most associated with SPON2 in TNBC were screened by Pearson correlation analysis from GEO databases GSE76275 and GSE103091 for GO and KEGG analysis. The biological processes most relevant to SPON2 in the GSE76275 dataset include cell adhesion, collagen fibril organization, angiogenesis, extracellular matrix organization, cell–cell adhesion, and positive regulation of cell migration. In addition, the cellular components most associated with SPON2 are extracellular region, extracellular matrix, extracellular space, endoplasmic reticulum lumen, basement membrane, and collagen trimer. The molecular functions most associated with SPON2 are extracellular matrix structural constituent, integrin binding, collagen binding, extracellular matrix structural constituent conferring tensile strength, heparin binding, and calcium ion binding. The most relevant signaling pathways for SPON2 are focal adhesion, ECM-receptor interaction, protein digestion and absorption, PI3K-Akt signaling pathway, human papillomavirus infection, proteoglycans in cancer. The biological processes, cellular components, molecular functions, and signaling pathways associated with SPON2 in the GSE103091 dataset are similar to those in the GSE76275 dataset (**Figure 2**).

### 3.3 Successful construction of SPON2 knockdown MDA-MB-231 cells

**Figure 3:**
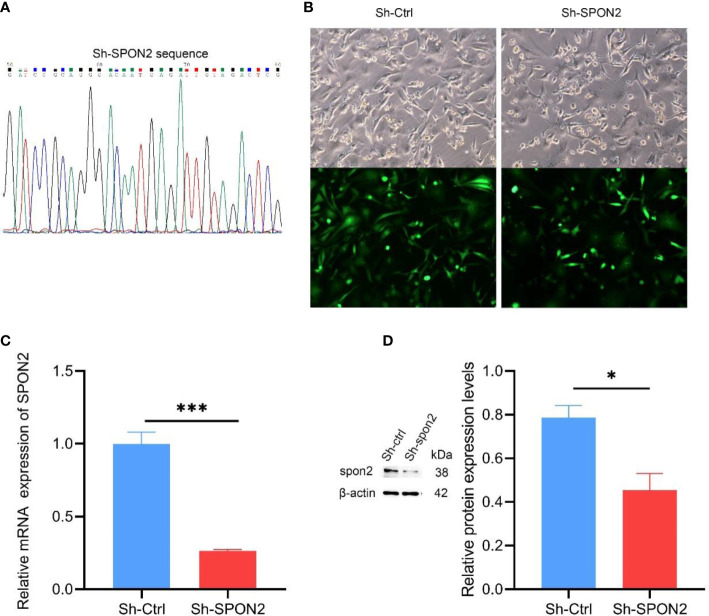
SPON2 knockdown MDA-MB-231 cells were successfully constructed. **(A)** The Sh-SPON2 plasmid was sequenced with the same design sequence. **(B)** Green fluorescence detected by fluorescence microscope, transfection efficiency > 95%. Magnification times: ×100. **(C)** The qPCR assay showed that the SPON2 knockdown group (Sh-SPON2) had lower SPON2 mRNA than the control virus group (Sh-Ctrl). **(D)** Western Blot assay showed that SPON2 protein expression was lower in the SPON2 knockdown group (Sh-SPON2) than in the control virus group (Sh-Ctrl). *P < 0.05, ***P < 0.001.

To investigate the function of SPON2 in TNBC, we constructed ShRNA-SPON2 lentiviral vector (**Figure 3A**). We knocked down MDA-MB-231 cells with high SPON2 expression with ShRNA-SPON2 lentivirus and used ShRNA-Ctrl virus as a control group to exclude the effect of lentivirus on the experimental results. The lentiviral vector used was characterized by a green fluorescent protein gene and puromycin resistance, and the successfully transfected cells expressed green fluorescence normally and were resistant to puromycin. Fluorescence photography showed successful introduction of the interfering sequence into the genome of MDA-MB-231 cells **Figure 3B**. We then treated the transfected cells with puromycin and used untransfected lentiviral MDA-MB-231 null cells as a reference, and the screening was completed when the null cell group was more than 90% dead. The qPCR and Western blot results showed that the expression of SPON2 in the sh-SPON2 group was significantly lower than that in the Sh-Ctrl group (**Figures 3C, D**).

### 3.4 SPON2 silencing inhibits TNBC cell proliferation, clone formation, migration, and invasion and promotes apoptosis

**Figure 4:**
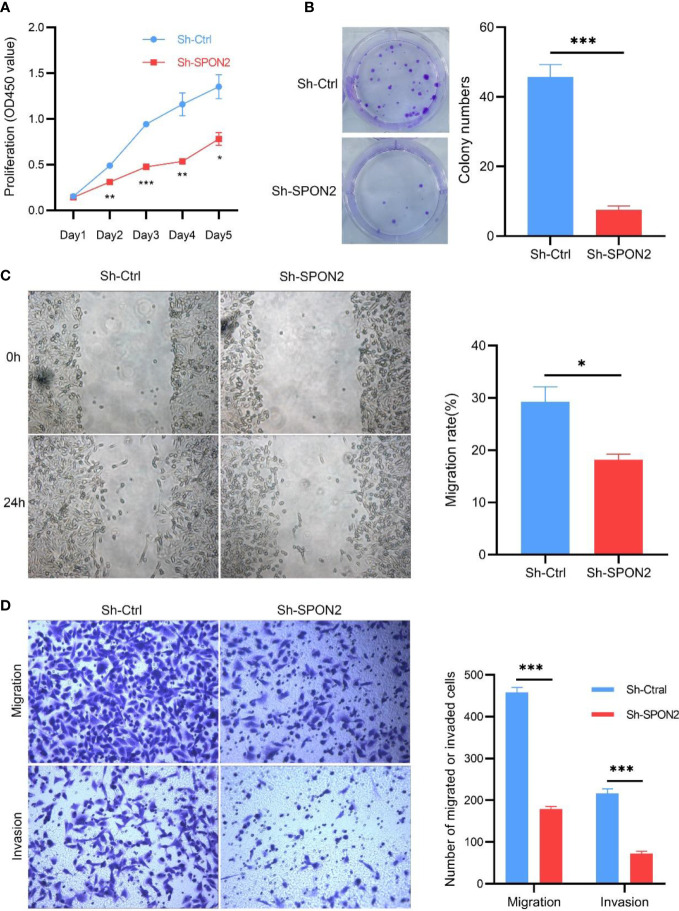
Knockdown of SPON2 inhibits proliferation, clone formation, migration, and invasion of MDA-MB-231 cells. **(A)** CCK8 showed that SPON2 knockdown inhibited the proliferative capacity of MDA-MB-231 cells. **(B)** Clonogenesis assays showed that SPON2 knockdown inhibited the clonogenic ability of MDA-MB-231 cells. **(C)** Scratch assay showed that SPON2 knockdown inhibited the migratory ability of MDA-MB-231 cells. Magnification times: ×100. **(D)** Transwell migration and invasion assay showed that SPON2 knockdown inhibited the migration and invasion ability of MDA-MB-231 cells. Magnification times: ×200. *P < 0.05, ***P < 0.001.

The CCK-8 assay was used to detect changes in MDA-MB-231 cell proliferation after SPON2 knockdown. Knockdown of SPON2 significantly reduced proliferation in the Sh-SPON2 group compared with the Sh-Ctrl group, **Figure 4A**. Flat-plate clonogenesis assay was used to detect changes in clonogenesis of individual MDA-MB-231 cells after SPON2 knockdown, and the results showed that SPON2 knockdown inhibited the clonogenic ability of MDA-MB-231 cells, **Figure 4B**.

**Figure 5:**
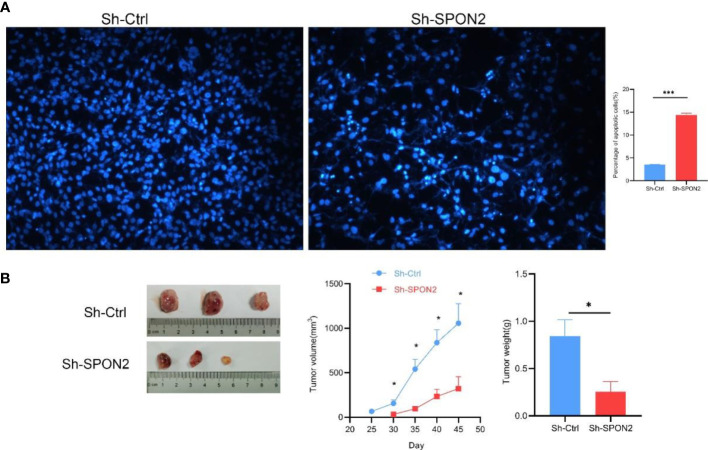
Knockdown of SPON2 promotes apoptosis of MDA-MB-231 cells and inhibits subcutaneous tumorigenesis in nude mice. **(A)** Hoechst apoptosis assay shows that knockdown of SPON2 increases apoptosis in MDA-MB-231 cells. Magnification times: ×100. **(B)** Inhibition of subcutaneous tumorigenic capacity of MDA-MB-231 cells in nude mice after SPON2 knockdown. *P < 0.05, ***P < 0.001.

Invasion and metastasis are important events in tumor progression. Therefore, we investigated the effect of SPON2 on TNBC cell invasion and migration using scratch assays, transwell migration and invasion assays. The results of scratch assay and transwell migration showed that knockdown of SPON2 could attenuate the migration ability of TNBC cells. Transwell invasion results showed that knockdown of SPON2 could attenuate the invasion ability of TNBC cells. **Figures 4C, D**. Hoechst apoptosis assay showed that knockdown of SPON2 increased the apoptosis of TNBC cells (**Figure 5A**).

### 3.5 Inhibition of subcutaneous tumorigenic capacity of MDA-MB-231 cells in nude mice after SPON2 knockdown

We injected the knockdown group Sh-SPON2 and the control group Sh-Ctrl cells subcutaneously into nude mice, and the results showed that SPON2 knockdown inhibited the tumorigenic ability of TNBC cells subcutaneously in nude mice (**Figure 5B**).

### 3.6 High-throughput transcriptome RNA sequencing reveals potential molecules and signaling pathways associated with SPON2 in TNBC

To explore the changes in the transcriptome of TNBC cells after SPON2 knockdown to find potential molecules and signaling pathways associated with SPON2, we performed high-throughput transcriptome sequencing on the knockdown group Sh-SPON2 and control group Sh-Ctrl cells.

**Figure 6:**
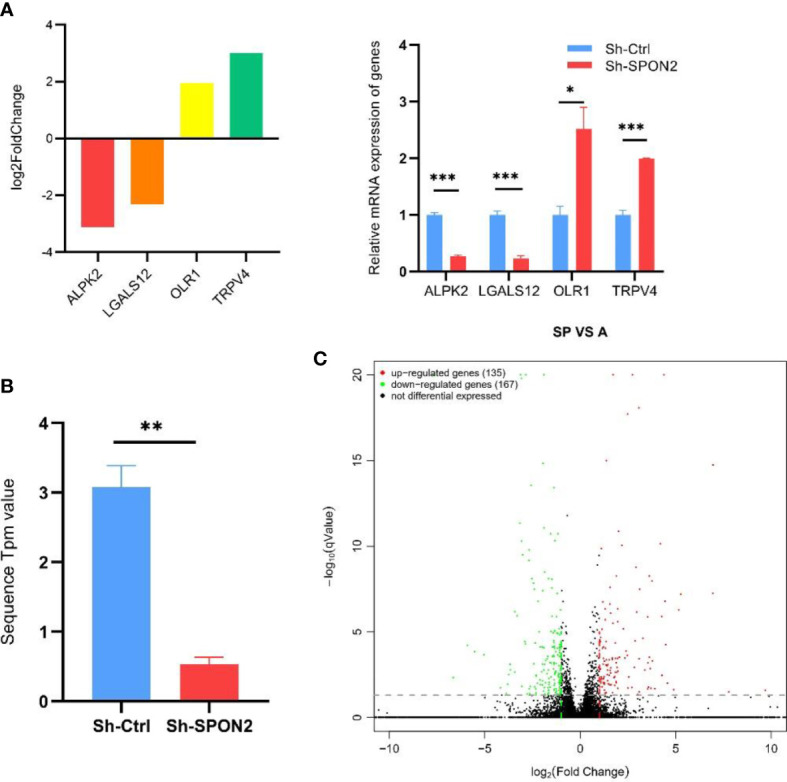
Transcriptome sequencing after knockdown of SPON2 in MDA-MB-231 cells. **(A)** Four transcriptome sequencing differential genes were randomly selected for qPCR validation, and the results showed that qPCR and transcriptome sequencing validated the differential gene expression trends in agreement. **(B)** Transcriptome sequencing showed that the SPON2 knockdown group (Sh-SPON2) had lower SPON2 expression than the control virus group (Sh-Ctrl). **(C)** The transcriptome sequencing volcano map showed that knockdown of SPON2 resulted in upregulation of 135 genes and downregulation of 167 genes. *P < 0.05, **P < 0.01, ***P < 0.001.

We randomly selected four transcriptome sequencing differential genes for qPCR validation, and the results showed that qPCR and transcriptome sequencing validated the differential gene expression trends in agreement (**Figure 6A**).

Transcriptome sequencing showed that SPON2 expression was reduced in the knockdown group Sh-SPON2 (**Figure 6B**).

The volcano plot showed that 135 genes were up-regulated and 167 genes were down-regulated after SPON2 knockdown (**Figure 6C**).

### 3.7 Transcriptome sequencing enrichment analysis

GO and KEGG analyses were performed based on the above differential genes. The biological processes most associated with SPON2 among expression up-regulated genes include synapse assembly, cell adhesion, biological adhesion, homophilic cell adhesion *via* plasma membrane adhesion molecules and calcium-dependent cell–cell adhesion *via* plasma membrane cell adhesion molecules, synapse organization. In addition, the cellular components most associated with SPON2 are plasma membrane part, integral component of plasma membrane, intrinsic component of plasma membrane, cell periphery, plasma membrane, and collagen type IV trimer. The molecular functions are phenanthrene 9,10-monooxygenase activity, ketosteroid monooxygenase activity, trans-1,2-dihydrobenzene-1,2-diol dehydrogenase activity, oxidoreductase activity, acting on CH-OH group of donors, alcohol dehydrogenase (NADP+) activity, alditol:NADP+ 1-oxidoreductase activity.

The biological processes most associated with SPON2 among expression down-regulated genes include antigen processing and presentation of peptide or polysaccharide antigen *via* MHC class II, T-cell costimulation, antigen processing and presentation of exogenous peptide antigen *via* MHC class II, lymphocyte costimulation, antigen processing and presentation of peptide antigen *via* MHC class II, acute-phase response. In addition, the cellular components most associated with SPON2 are MHC class II protein complex, MHC protein complex, COPII-coated ER to Golgi transport vesicle, endoplasmic reticulum part, clathrin-coated endocytic vesicle membrane, and transport vesicle. The molecular functions are MHC class II receptor activity, peptide antigen binding, lipoprotein lipase activity, dioxygenase activity, ornithine decarboxylase regulator activity, and chemoattractant activity.

**Figure 7:**
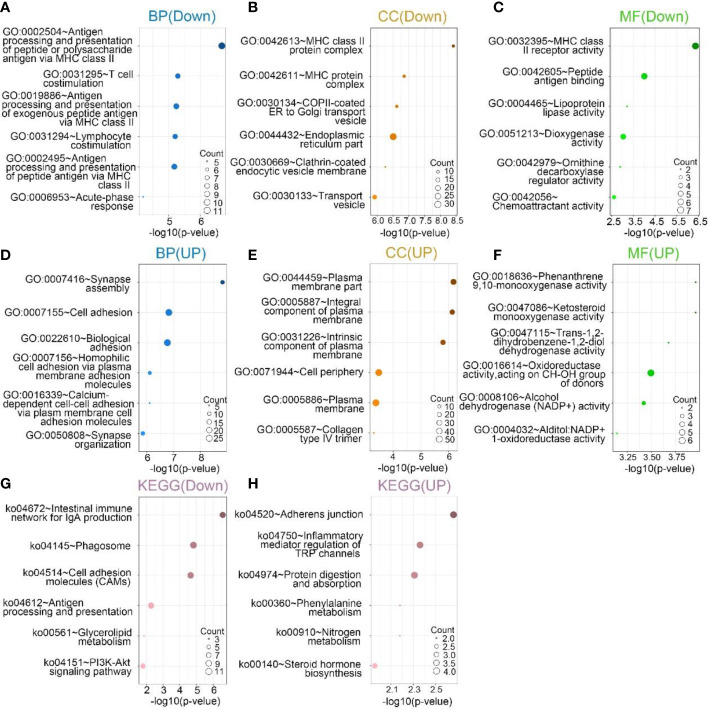
**(A–C, G)** In the sequencing downregulation group, the top 6 biological processes (BPs), cellular components (CCs), and molecular functions (MFs), Kyoto Encyclopedia of Genes and Genomes (KEGG) pathway analysis most related to SPON2. **(D–F, H)** In the sequencing upregulation group, the top 6 biological processes (BP), cellular components (CCs), and molecular functions (MFs), Kyoto Encyclopedia of Genes and Genomes (KEGG) pathway analysis most related to SPON2.

According to KEGG analysis, among the up-regulated pathways, the most relevant signaling pathways for SPON2 are adherens junction, inflammatory mediator regulation of TRP channels, protein digestion and absorption, phenylalanine metabolism, nitrogen metabolism, and steroid hormone biosynthesis. Among the down-regulated pathways, the most relevant signaling pathways for SPON2 are intestinal immune network for IgA production, phagosome, cell adhesion molecules (CAMs), antigen processing and presentation, glycerolipid metabolism, and PI3K-Akt signaling pathway (**Figure 7**).

**Figure 8:**
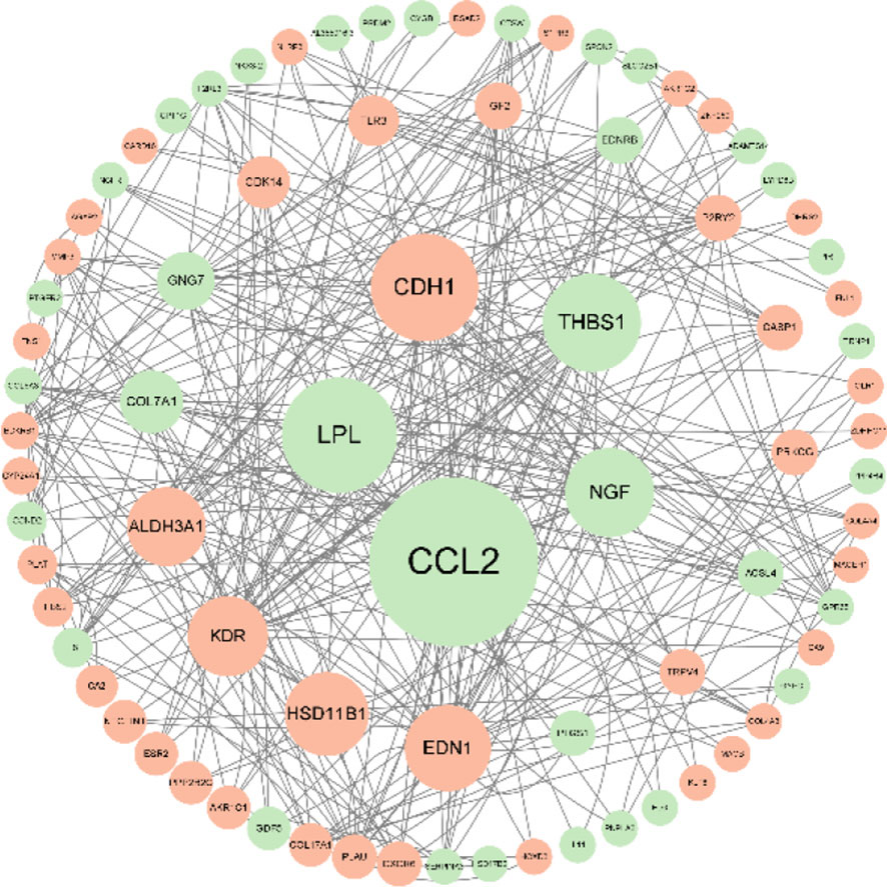
After knocking down SPON2, PPI network analysis of differential genes showed that CCL2, LPL, CDH1, THBS1, and NGF scored higher and were important key proteins. The larger the circle area, the higher the betweenness score; green represents the down-regulated genes and represents the up-regulated genes.

Psychophysiological interaction (PPI) network analysis was performed on the differential genes to produce PPI network maps. The CytoNCA plug-in was used to calculate betweenness (BC), and the analysis showed that CCL2, LPL, CDH1, THBS1, and NGF scored high and were important key proteins (**Figure 8**).

### 3.8 PI3K-AKT pathway

**Figure 9:**
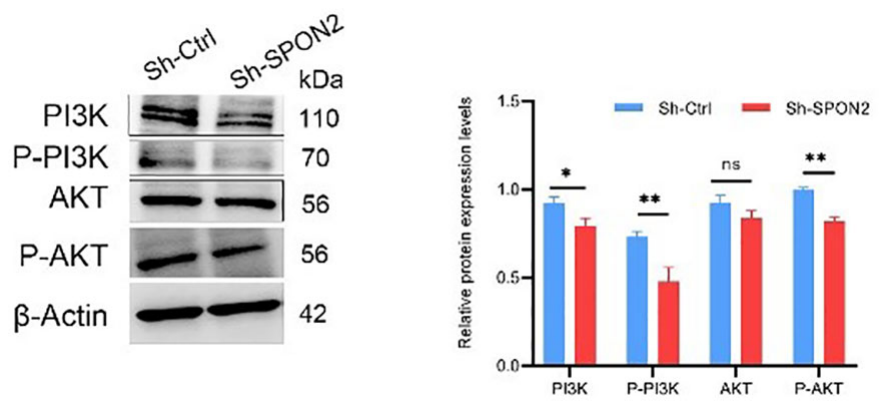
Western Blot assay showed that PI3K-AKT pathway expression was down-regulated after knockdown of SPON2. *P < 0.05, **P < 0.01, ns, no significance.

KEGG analysis of GEO database GSE76275 and GSE103091 revealed that SPON2 expression in TNBC was associated with PI3K-AKT pathway. After SPON2 knockdown, transcriptome sequencing showed that the expression of PI3K-AKT pathway was down-regulated in TNBC cells, suggesting that SPON2 may play a role in TBNC through the PI3K-AKT pathway. We then investigated the changes of PI3K-AKT pathway expression in knockdown Sh-SPON2 and control Sh-Ctrl cells using Western blot experiments. The results showed that the expression of PI3K-AKT pathway was down-regulated after SPON2 knockdown in TNBC cells (**Figure 9**).

## 4 Discussion

In this study, we demonstrated by immunohistochemical experiments that SPON2 protein expression was higher in TNBC tissues than in normal tissues adjacent to cancer and breast fibroadenoma. Analysis using the GEO database GSE76275 dataset showed that SPON2 mRNA expression was higher in TNBC than in non-TNBC. Cellular qPCR experiments showed that SPON2 mRNA expression was higher in TNBC cells MDA-MB-231 than in non-TNBC cells and normal breast cells. Some studies have shown that TNBC is more prone to invasion, metastasis, and recurrence compared with the other types of breast cancer (19, 20). In normal breast, non-TNBC and TNBC, SPON2 expression increased with increasing malignancy. Kaplan-Meier Plotter database analysis showed that the higher the SPON2 expression in TNBC, the worse the prognosis. It is suggested that SPON2 may play a pro-cancer role in TNBC.

To investigate the role played by SPON2 in TNBC, we successfully established SPON2 knockdown in TNBC cells with MDA-MB-231. Cellular experiments showed that knockdown of SPON2 expression significantly decreased the proliferation and clonogenic ability of TNBC cells, significantly inhibited cell migration and invasion ability, and increased the number of apoptotic cells. These results suggest that SPON2 expression is up-regulated in TNBC and can promote TNBC cell proliferation and invasion, suggesting that SPON2 may promote the occurrence and development of TNBC.

GO analysis of the GEO database GSE76275 and GSE103091 datasets revealed that the most relevant biological process for SPON2 is cell adhesion. KEGG analysis revealed that the most relevant signaling pathway for SPON2 is focal adhesion. Transcriptome sequencing after knockdown of SPON2 and GO analysis of differential genes revealed that biological processes such as cell adhesion, biological adhesion, homophilic cell adhesion *via* plasma membrane adhesion molecules and calcium-dependent cell–cell adhesion *via* plasma membrane cell adhesion molecules were up-regulated. Moreover, knockdown of SPON2 was followed by upregulation of the signaling pathway adherens junction. Metastasis of tumors is a complex process involving multiple steps and factors (21). In this process, altered cell adhesion capacity is a key element in the development of invasive metastasis of tumor cells (19). Knockdown of SPON2 may result in enhanced adhesion of TNBC cells, thereby inhibiting migration and invasion.

The PI3K/AKT/mTOR signaling pathway is a cell cycle-related signaling pathway that plays a key role in the regulation of cell proliferation, growth, survival, motility, and metabolism (22). In addition, aberrant activation of the PI3K/AKT/mTOR signaling pathway is frequently found in a variety of cancers (23). In recent years, the PI3K/AKT/mTOR signaling pathway has been recognized as an important signaling pathway in breast cancer (24). This signaling pathway is activated in up to 70% of breast cancers (25). We found that SPON2 was associated with the PI3K-AKT pathway in TNBC by KEGG analysis of the GEO database GSE76275 and GSE103091 datasets. Transcriptome sequencing showed that PI3K-AKT pathway was down-regulated after knockdown of SPON2. Western blot assay demonstrated that the expression of P-AKT and P-PI3K was down-regulated in TNBC cells after knockdown of SPON2. Therefore, downregulation of SPON2 expression may affect TNBC cell proliferation, migration, invasion, apoptosis, and tumorigenic ability *in vitro* by inhibiting the PI3K-AKT pathway.

Excess nitrogen in the body produces toxic ammonia, which can cause irreversible damage to the organism (26). The lipid synthesis provides essential substrate for energy metabolism and ingredients for cell membrane construction during the proliferation process, suggesting an indispensable role of lipid metabolism pathways in the genesis and development of cancer (27). After SPON2 knockdown, KEGG analysis showed that nitrogen metabolism was up-regulated and glycerolipid metabolism was down-regulated. In contrast, TNBC cell growth was inhibited following SPON2 knockdown, suggesting that these metabolic pathways may play a role and are interesting lines of research.

Transcriptome sequencing PPI protein network analysis revealed that CCL2 is the key protein. CCL2 gene expression was down-regulated in TNBC after knockdown of SPON2.

CCL2 promotes tumor invasion and metastasis by recruiting mononuclear macrophages (28). It was found to be highly expressed in a variety of tumors such as gastric, breast, lung, and cervical cancers and correlated with poor patient prognosis (29–32). CCL2 has pro-tumor effects and CCL2 affects vascular endothelial cells through the JAK2-STAT5 and P38 mitogen-activated protein kinase pathways, regulating tumor vascularization and tumor metastasis (33). It can promote the secretion of MMP2 and MMP9 (34, 35), which, in turn, degrade the stroma to promote tumor cell metastasis (36). Silencing CCL2 was found to inhibit the development of TNBC by blocking tumor stem cell self-renewal and M2-type macrophage recruitment (37). Therefore, knockdown of SPON2 may cause downregulation of CCL2 expression, thereby affecting TNBC cells.

In summary, our study showed that SPON2 was up-regulated in TNBC and correlated with poor patient prognosis. Knockdown of SPON2 inhibited TNBC cell proliferation, clone formation, migration, invasion, and tumorigenic ability and promoted apoptosis. SPON2 may play a role in TNBC through cell adhesion, PI3K-AKT pathway. The possible role of SPON2 in tumors through glycerolipid and nitrogen metabolic pathways is a new direction in the study of SPON2 function. SPON2 may be a promising biomarker for the diagnosis and prognosis of TNBC and a potential therapeutic target for TNBC.

## Data availability statement

The raw data supporting the conclusions of this article will be made available by the authors, without undue reservation.

## Ethics statement

The studies involving human participants were reviewed and approved by Ethics committee of Sinopharm Dongfeng General Hospital, Hubei University of Medicine. Written informed consent for participation was not required for this study in accordance with the national legislation and the institutional requirements. The animal study was reviewed and approved by Experimental Animal Welfare Ethics Review Committee of Hubei University of Medicine.

## Author contributions

XH: Study design, Cell and animal experiments, the article writing. CS: Immunohistochemical test. JW: The data analysis, Manuscript review, Funding acquisition. All authors contributed to the article and approved the submitted version.
